# Promoting Intergenerational Justice Through Participatory Practices: Climate Workshops as an Arena for Young People’s Political Participation

**DOI:** 10.3389/fpsyg.2021.727227

**Published:** 2021-12-06

**Authors:** Marit Ursin, Linn C. Lorgen, Isaac Arturo Ortega Alvarado, Ani-Lea Smalsundmo, Runar Chang Nordgård, Mari Roald Bern, Kjersti Bjørnevik

**Affiliations:** ^1^Department of Education and Lifelong Learning, Norwegian University of Science and Technology, Trondheim, Norway; ^2^Department of Design, Norwegian University of Science and Technology, Trondheim, Norway; ^3^Trondheim Cathedral School, Trondheim, Norway; ^4^Heimdal High School, Trondheim, Norway; ^5^Trøndelag County Council, Trondheim, Norway

**Keywords:** children, youth, participation, participatory methods, climate, intergenerational justice, citizenship, children’s rights

## Abstract

In the fall of 2019, Trøndelag County Council, Norway, organized a Climate Workshop for children and youth. The intention of the workshop was to include children’s and youth’s perspectives as a foundation for a policy document titled “How we do it in Trøndelag. Strategy for transformations to mitigate climate change”. The workshop involved a range of creative and discussion tools for input on sustainable development and climate politics. In this article, we aim to (1) describe and discuss innovative practices that include children and youth in policymaking related to climate action, and (2) discuss the theoretical implications of such policymaking in relation to children’s rights, young citizenship, and intergenerational justice. We employ a generational framework and perceive climate politics as inherently ingrained in intergenerational justice, where no generation has a superior claim to the earth’s resources, yet power is unfairly concentrated and accumulated among adult generations. We draw on contributions by various stakeholders involved: Two young workshop participants, two county council policymakers, and an interdisciplinary team of researchers from Childhood Studies and Design.

## Introduction

In the fall of 2019, Trøndelag County Council, Norway, organized a Climate Workshop for children and youth aged 10–18 years. The backdrop for the event was the growing global movement where children and young people demonstrate against the lack of political will to realize the goals set out in the Paris Agreement. In Trøndelag, this led to a school strike in Tordenskioldsparken on March 22, 2019, when around 3,000 students demanded political action to ensure a more sustainable future. This mobilization triggered local politicians to invite children and youth into the process of preparing a new Climate Strategy for Trøndelag County. Politicians sought not only to include, but also promote ownership of an environmental strategy among the region’s youth. In collaboration with researchers, Trøndelag County Council designed the Climate Workshop where children and youth were asked about their experiences with climate issues in their everyday lives as well as their visions for a sustainable future and ways to achieve this vision. The participants shared both frustrations and solutions for climate politics. Thirty-eight children and youth participated in the two-day workshop, which included a range of creative and discussion tools deriving from Participatory Design and participatory methods within Childhood Studies.

In this article, we aim (1) to discuss the inclusion of children and youth in policymaking related to climate action, and (2) to discuss the theoretical implications of such policymaking in relation to children’s rights, young citizenship, and intergenerational justice. We employ a generational framework and perceive climate politics as inherently engrained in intergenerational justice, where no generation has a superior claim to the earth’s resources, yet power is unfairly concentrated and accumulated among adult generations. We draw on contributions by the various stakeholders involved: two young workshop participants, two county council policymakers, and an interdisciplinary team of researchers from Childhood Studies and Design. The article thus is inclusive of multiple viewpoints on potentials and challenges when including children and youth in political processes across research disciplines, sectors, and generations. However, the article has an ‘unitary voice’ where authors’ ownership of ideas and arguments remain obscured. We contributed on equal terms to avoid ‘othering’ of non-academic authors. Elsewhere, we have taken advantage of multivocal co-authorship, allowing tensions to emerge (see Ursin et al., in review^1^).

The article is structured as follows: first, we explore how intergenerational justice can be understood and approached in climate politics. In the “Materials and Methods” section, we describe the methodology and methods of the Climate Workshop. In results, we first illustrate the material generated in the workshop before we describe themes identified in the assessment of the workshop. In the discussion, we examine some strengths and weaknesses with the Climate Workshop, and critically reflect on the degree to which participatory workshops with children and youth are useful in enhancing their participatory rights, sense of citizenship and intergenerational justice.

## Intergenerational Justice and Climate Legacy

Emission-generating activities grant the present generation numerous benefits—e.g., infrastructure, industrial goods, food, transportation—while the effects are likely to be harmful for generations to come (Meyer, 2012). Due to the time lag of anthropogenic climate change, an increasing number of theorists within Law and Philosophy call for new legal principles that recognize this intergenerational connection among human societies and articulate the rights and corresponding duties that underpin intergenerational equity (Weston and Bach, 2009). The legal principle of intergenerational justice concerns ‘justice between generations,’ a transgenerational respect for the rights and fulfillment of duties vis-à-vis future and past generations (Meyer, 2012). It concerns intergenerational conflict of interests, seeks to solve inter-temporal distributive questions (Barry, 2012/1999), and calls for temporal solidarity across past, present, and future generations (Weston and Bach, 2009). Intergenerational justice bears many similarities with social justice though a class dimension is substituted with a generational dimension. Although valid in all matters concerning distribution of resources, it is especially fruitful in environmental politics, anthropogenic climate change, and global warming, as intergenerational equity is key to sustainability (Barry, 2012/1999). The dilemma of intergenerational justice is its inter-temporality, where distributive justice entails the ability to take into consideration both the concrete and lived present and the uncertain future.

According to Meyer (2012), environmental politics solicits global intergenerational distributive justice:

Assuming that future people will suffer serious harm in terms of the violation of their basic rights when temperatures rise above a certain level and, further, that currently living people can hinder such temperature rise by limiting their emissions to a certain amount, a global cap on emissions is required for currently living people to be able to fulfill their minimal duties of justice vis-à-vis future generations (xix).

Rawls’ principle of “just savings” is of importance, where parties must agree to a savings principle that ensures that each generation receives its due from its predecessors and does its fair share for those to come (Rawls, 2012/1971, p. 18). It is futile to agree as to what ‘just savings’ encompasses. To solve this, Parfit (2012/1984) suggests that “[w]hen we cannot ask for someone’s consent, we should instead ask whether this person would later regret what we are doing” (p. 45). Because of time’s arrow, we cannot do anything to make people in the past better off than they were (Barry, 2012/1999, p. 197), encapsulating the dilemma of reciprocity-based intergenerational justice between present and future generations. As Gardiner (2012/2003) notes, there is a generational asymmetry, involving an asymmetric independence of interests (interests of earlier groups are independent of interests of later groups) that rules out intertemporal exchange for mutual advantage. This form of indirect reciprocity is what Weston and Bach (2009) refer to as the “stewardship model.”

Intergenerational justice can also be seen as a transgenerational global social contract that is founded on human solidarity. According to this perspective, the “common heritage” of earth’s natural resources, freshwater systems, oceans, atmosphere, and outer space all belong to generations in an intertemporal partnership (Weston and Bach, 2009). Time is not seen as a three-point linear order of past, present, and future, but humanity is rather perceived as consisting of transgenerational communities with lifetime-transcending interests (Campos, 2018). Responsibilities toward non-overlapping generations will ensure the preservation of the cultural identity of communities over time and ensure survival of the planet and all life therein.

Regarding global climate politics and policymaking, an intergenerational perspective has been vital from the onset. The first world conference to make the environment a major issue, the United Nations Conference on the Environment in Stockholm in 1972, included an intergenerational approach in the final Declaration: “To defend and improve the human environment for present and future generations has become an imperative goal for mankind” (United Nations, 1972, section 6). In the so-called Brundtland Report, the United Nations (1987) further explicated the connection between intergenerationality and climate politics, as it is deeply embedded in the concept of sustainable development, that is, our “ability to make development sustainable to ensure that it meets the needs of the present without compromising the ability of future generations to meet their own needs” (p. 43). Likewise, the United Nations’ Education for Sustainable Development initiative now also seeks to integrate the values and practices of sustainable development into all aspects of education, envisaging children and young people as powerful agents of change (Walker, 2017).

Within the literature on intergenerational justice, discussions on definitions of ‘future generations’ are manifold. Weston and Bach (2009) draw on Boulding’s (1978) conceptualization of the ‘200-year present’ as a continuously moving moment, a sort of fluid present, stretching 100 years in either direction from the current moment. Future generations, they conclude, are the three and a half generations of persons that exist from this day forward, including children (i.e., persons under 18) because “they usually are poorly positioned to determine their future and thus, like future generations, require others to represent their interests” (ibid: 18, see also Gardiner, 2012/2003). Thus, children and youth hold an important position as betwixt-and-between. According to Davies et al. (2016), children’s position in regards to climate change, politics and intergenerational justice is marked by four factors: (1) Children are vulnerable to climate change and climate induced effects due to their physiology and immature immune systems, lack of access to financial resources and means of transit, high care needs, and dependence on adults; (2) Children and unborn generations will bear the brunt of of long-term climatic changes; (3) Children are our closest connection to future generations; and (4) Children’s views are traditionally excluded from legal and political debates concerning climate politics. The Climate Workshop described below was aimed at countering this by including children’s and youth’s perspectives in the shaping of regional climate policy.

## Materials and Methods

### Methodological Approach: Participatory Design

The Climate Workshop was not a research project, but an initiative organized by Trøndelag County Council and informed by participatory methods originating within two different scholarly traditions: Participatory Design and Childhood Studies. Participatory Design has its roots in Scandinavian countries, where it served to democratize the decision-making process in factories by including workers—as the affected group— in the formulation of solutions regarding the use of new technologies that could result in job displacements (Kensing and Greenbaum, 2012). The original idea behind Participatory Design was to minimize the negative effects on workers by co-producing solutions that included their perspectives and required their involvement in the implementation. Participatory Design processes are about opening decision-making and solution-enactment from the perspectives of implicated actors. Participatory methods are used to engage people in inquiring about problems and thinking about solutions. Participation thus requires the active engagement of participants as co-creators of solutions (Sanders and Stappers, 2008).

In Childhood Studies, participatory research is based on a view of children as *social agents* (they hold valid knowledge) and *subjects of rights* (not objects). Research should be done *with* rather than *on* children (James and Prout, 1997). Traditional research has tended to underestimate the competencies of children and young people, often relying on adults to represent their perspectives (van Blerk and Ansell, 2007). In Childhood Studies, children and youth are recognized as experts of their own lives, having their own agendas and interests. Participatory research enables children and youth to express their perspectives and opinions freely whilst also ensuring their human rights (Ennew et al., 2009). Drawing on the UN Convention of the Rights of the Child (United Nations, 1989), Beazley et al. (2009) describe a rights-based approach as securing children and youth the right to the highest possible standards in work with them (Article 3.3), the right to provide opinions (Article 12), the right of expression with a medium of their own choice (Article 13), and the right to be protected from exploitation (Article 36). Translating into research and policymaking, this entails involving children and youth as participants, using methods that allow them to easily express their opinions, views, and experiences (not limited to verbal expressions), and protecting them from any harm that might result from their participation. Using methods that allow children and youth to express views in *a variety* of ways, not only verbal, is a central feature of participatory research within Childhood Studies (Ennew et al., 2009). Task-based and visual techniques are often presented as ‘child-friendly’ (Punch, 2002), enabling a more ‘direct’ expression of views. An important rationale for drawing on a range of methods is maximizing young participants’ willingness and ability to express views (Punch, 2002).

The Climate Workshop was inspired by the method for future workshops (Jungk and Müllert, 1997), a well-known method in Participatory Design with two main purposes: (1) Attainment of the vision of participants in a way that is respectful to their perspectives, and (2) legitimization of participants’ perspectives without the intrusion of ‘expert’ knowledge. Collective future envisioning by young people sought to gather and attest imaginary preferred futures from participants’ expectations through collectively drawn or written stories about future everyday life. We found this method suitable for young citizens because it allows for exploration of what they see as main issues, what they want, and what they are willing to do. Alminde and Warming (2020) discuss the application of future workshops as democratic research with children and youth and regard it as “a creative participatory process rather than merely a collection of opinions and data” (ibid, p. 444). Following the suggestions by Brandt et al. (2012, p. 152), we decided to include the everyday life perspective as it is the present circumstance in which climate change is recognized as a problem and arguably where children and youth have the most room for agency. The future workshops method consisted of three steps: (1) Critique, where participants express what they understand as the problem; (2) Fantasy, where participants create a desirable or idealized future situation; and (3) Realization, where participants create an action plan. These steps were carried out through an overall focus on everyday experiences of climate related challenges on Day 1 and a focus on visions for a sustainable future on Day 2 (elaborated below). Realization was addressed both days by inviting participants to suggest solutions for identified challenges and ways to achieve their visions for an ideal future.

**PHOTO 1:**
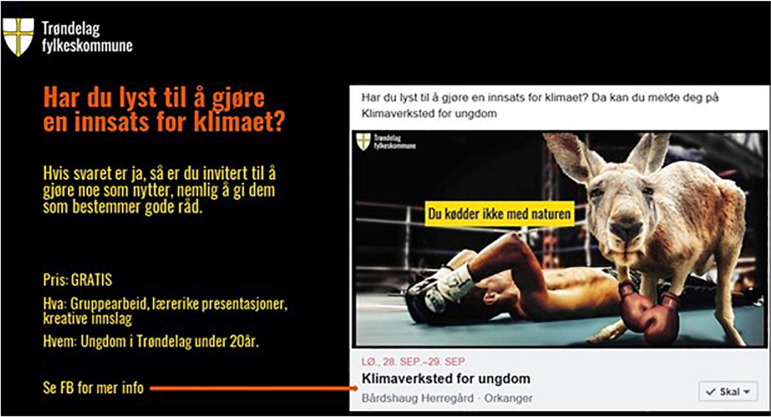
Open invitation for Climate Workshop for youth.

For the recruitment of participants, open invitations (see example in Photo 1) aimed at 13–19-year-olds were created together with Trøndelag youth county committee and distributed through messaging boards in high schools and social media (Snapchat, Instagram, and Facebook). The county council also sent an invitation to an umbrella organization of 40 local youth organizations and the School Student Union. Young people were invited to ‘make an effort for climate’ through giving ‘advice to those in charge.’ A Facebook event informed potential participants that the results from the workshop would be used in the development of a new strategy for climate mitigation in Trøndelag. This was repeated in the welcoming speech of the Climate Workshop. Participation was free of charge and included an overnight stay at the hotel where the workshop would be held on a weekend in September 2019; the county council aimed to ensure that finances would not be a barrier for participation. Despite primarily targeting youth above the age of 13, younger children who expressed an interest in participating were also welcomed. Thirty-eight children and youth between the ages of 10 and 18 signed up for the workshop. The majority was aged 13–18 years while three participants were 10–12-year-olds. Fifteen participants were from the city of Trondheim and the rest from other areas in the county. Most participants were girls (25 girls, 12 boys and one participant who identified as non-binary), and half of the participants were active in organizations such as political councils, political parties, or environmental groups.

The time frame for the activities in the workshop was limited, with four hours on Day 1 and three hours on Day 2. Before the activities started, there was a lunch with short speeches from the organizers and youth activists. The participants were organized in seven groups of four to six people. The groups were sorted by age–three groups of 15–18-year-olds gathered in one room, and four groups of 10–15-year-olds in another. We reasoned that although communication across a wider age span could be productive, it could make participation a daunting experience for the younger ones due to unbalanced power dynamics (Langevang, 2009). Each group was assigned a youth facilitator with a background from Trøndelag’s youth county committee and the organization UngEnergi. They were briefed to ensure a good understanding of their tasks: To facilitate discussion, attend to power dynamics in the groups, and if possible, to observe and take notes about how participants worked together and solved tasks. The county council provided various art supplies and materials, including large cardboard posters, paper and permanent markers in assorted colors, pens, rulers, scissors, post-its in different sizes and colors, and decorative stickers.

**FIGURE 1:**
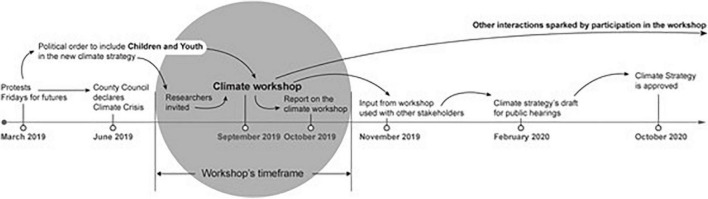
Diagram of the workshop’s time horizon.

Lorgen and Ursin wrote a report of the results after the workshop, while a youth facilitator read a draft and provided feedback. The county council published the report (Lorgen and Ursin, 2019). Input from the workshop was presented at workshops arranged by the county council with other (adult) stakeholders during the fall of 2019. A hearing draft for the climate transition strategy, as well as the report (Lorgen and Ursin, 2019) and input from the Climate Workshop were presented at a youth county council meeting in November 2019. The final strategy is based on the Climate Workshop report in addition to input from other stakeholders and knowledge from international climate research and national expert papers on how to tackle climate change in Norway (Trøndelag County Council, 2020). Although the workshop—as an event—had a limited timeframe, events around it went beyond its time horizon (see Figure 1).

#### Day 1 – Description of Activities

Day 1 activities focused on everyday experiences with climate related challenges and possible solutions. Each participant was asked to spend 10 min on writing a list of climate related issues and challenges they consider central to a political strategy. Participants then discussed their lists in groups. The individual activity was designed to provide space and time to articulate viewpoints before entering a group dynamic, thus working to include views from all participants and prevent some group members from dominating the discussion. Groups were then tasked with a ranking-activity, where they made a list of five numbered issues with a written explanation for why the issue was included in the priority list. Lists were written on large sheets of paper, and various tools and materials were made available to invite visual and creative solutions of the task. With the introduction of this visual aspect, we saw group dynamics evolve with some groups spreading out on the floor, actively using the tools and space available, contributing to a relaxed but energetic atmosphere. After lists were completed, the rankings were displayed and presented by participants to facilitate exchange of views across the groups.

**PHOTO 2:**
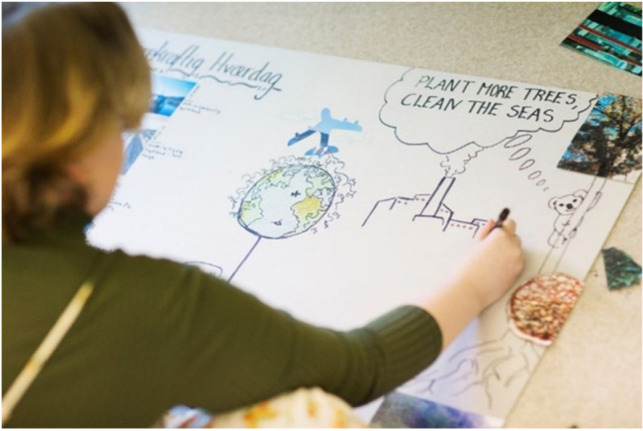
Collage about challenges and opportunities in sustainable everyday life.

In preparation for the workshop, participants were asked to select three to five photos, screen shots, or news clippings that illustrate climate related challenges or opportunities. The images were meant both as a way of inviting reflection on the topic in advance and provide visual material for the workshop. Participants showed their images and explained what they represented for their group. After everyone had presented their images, the groups were tasked with making a collage about “challenges and opportunities in sustainable everyday life,” using a large poster, tools and materials, and printed images (see Photo 2). Day 1 concluded with a final task, where the groups, based on discussions and resulting collage, were invited to consider where, when, and in which situations sustainable living is difficult to identify and in which areas where youth, families, or others need support. Each group again ranked important challenges and ideas for addressing them. Collages and ranking lists were displayed, and participants walked around and looked at each other’s work after the final task for the day was completed to facilitate a flow of ideas across groups and invite reflection before the final workshop day.

#### Day 2 – Description of Activities

Day 2 centered on fantasy and realization. In the first activity, participants were asked to create a vision of life in Trøndelag 10 years into the future. They could write a story or draw a comic strip about a day in the future life of a young person like themselves. It was made clear that it should be an ideal future world – without climate crisis – and that they were free to imagine any solution possible – even those that require technology that is not currently available. Participants were given questions to support their vision, such as: *How do you and your family live in the future? How do people live in a more sustainable way? What would you change to make everyday life more sustainable in 10 years?* Participants presented their desired scenario, illustrating their hopes for the future. Presentations concluded the activity.

**PHOTO 3:**
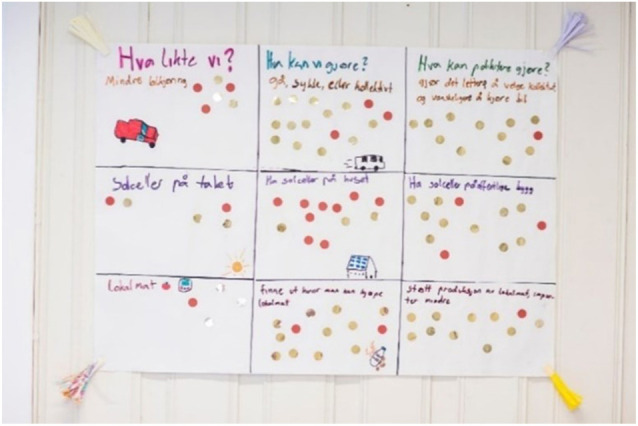
List of objectives and evaluations of degree of difficulty.

The remaining tasks were designed to address realization by inviting participants to create a plan for action. The groups first wrote down things they liked about the different future visions presented and some objectives (what do we want?). They then made a list of what they *themselves* could do and what *others*, like schools, institutions, and politicians could do to realize these objectives. Lists were displayed and participants were asked to walk around, read the suggestions, and classify them as easy or difficult using gold and red stickers (see Photo 3). Each participant then selected one ‘easy’ and one ‘difficult’ suggestion that they liked by writing these suggestions on post-its and sticking them onto two boards, one marked ‘easy’ and one ‘difficult.’ Participants were invited to indicate which actions they would be willing to take and which ones they expect others to do. Through this and the previous day’s ranking lists, political issues became evident as the participants indicated what they would put forward and what they were expecting politicians to carry out. The use of stickers and post-its to identify easy and difficult solutions also offered insight into participants’ expectations toward themselves and others.

The activities from the workshop provided a set of visual objects, which was the primary input for work on the Climate Strategy (Table 1).

**TABLE 1:** Summary of visual objects available for analysis.

Future workshop step	Result	Quantity
Critique	Priority lists	7
	Collages depicting problems	7
	List of challenges	7
Fantasy	2030 stories	7
Realization	Lists of actions	7
	List of easy vs. difficult actions	2

At the end of Day 2, the participants and youth facilitators were asked to offer feedback regarding the workshop, including both positive aspects and areas for improvement. We coded and categorized the feedback and discussed it. In addition, we exchanged reflection notes detailing their experiences with the workshop. Based on these materials, Ursin suggested five main themes that emerged from participant feedback and author reflections. We address these five themes in the discussion section after offering insight into the results of the workshop itself below.

## Results

Overall, the expectations of participants are centered on local, communal, and shared use of resources. At the same time, plastics and pollution from overconsumption are concerns. Table 2 presents priorities.

**TABLE 2:** Summary of main priorities identified.

Priorities:
**Renewable energy:** cheap and green replacement of fossil fuels. **Transportation:** public transport, cheap and electric options. **Environment:** conservation, biodiversity, and nature management, stop deforestation. **Plastics:** waste that pollutes forests and oceans, use of alternatives like wood to reduce the use of plastics. **Local production:** less importation and exportation of goods. **Sustainable consumption:** reuse versus overconsume. **State regulations:** policies for production and consumption and the environment. **Knowledge to youth and children:** inclusion in educational content **Reduction of CO_2_:** from manufacture and transportation. **Food:** waste as a problem and local production as a solution.

**FIGURE 2:**
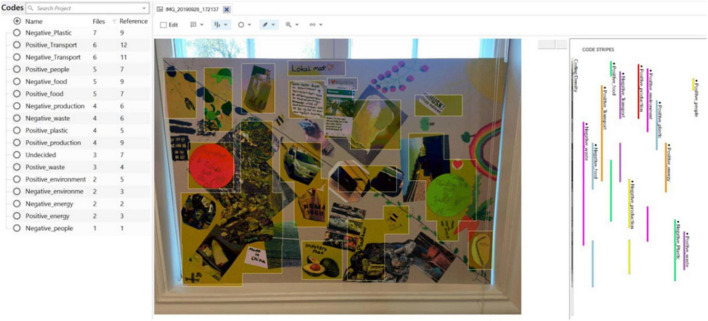
Snapshot of the visual categorization of one collage using the software NVivo 20.

Negative and positive connotations of materials were identified from the collages presenting opportunities and challenges in everyday life. Images and texts used in the collages were visually categorized under themes and connotations—positive or negative—resulting in 17 categories (see Figure 2). Initially the categorization included eight themes, ‘energy,’ ‘transportation,’ ‘plastics,’ ‘food,’ ‘waste,’ ‘production,’ ‘people,’ and ‘environment.’ The theme ‘people’ was renamed ‘consumption.’ Furthermore, ‘waste,’ ‘environment,’ and ‘production’ were recategorized under ‘pollution,’ finally ‘energy’ was included in transportation as summarized in Table 3.

**TABLE 3:** Themes identified with negative and positive connotations in the collages.

Theme	Negative	Positive
Transportation	- Cars and airplanes - Fossil fuels	- Collective: buses, trains, bikes - Electricity from windmills and bioenergy
Food	- Meat – importation - Waste	- Local – vegetarian - Composting
Consumption	Overconsumption	Reuse – reduction
Pollution	- Solid waste in nature (forests, oceans) - CO_2_	- Cleaning - Removal
Plastics	- Pollutant	- Replacement - Prohibition - Removal

Aside from the negative and positive connotations, we see in the future stories which material entities support the type of society that participants envisioned (Table 4). Expectations are about local production—such as wool—accompanied by technologies like windmills and solar cell panels in a thriving natural environment. The expected material supports were extracted from a visual analysis of the stories, coupled with the transcription of the text of the stories (6 out of 7).

**TABLE 4:** Summary of objects appearing in the stories.

Theme	Expected objects
Energy	Solar cell panels, plus-buildings, ^1^windmills, bioenergy, nuclear power plants.
Food	Vegetarian, homegrown, local, free-food refrigerators, ^2^insects, vegetables grown in windowsill.
Transportation	Free electric bicycles, bicycles, electric buses and trains, flying buses, drones.
Waste	Edible plates, environmental police, bio wax film to protect food.
Clothes and others	Made from local wool and hemp, bamboo or wooded toothbrush, hand-me-down clothes, reused clothes.
Housing	“Common garage,” kitchen with space for vegetable growing. Greenhouse for each house.
Education	“Climate and environment” course, history on climate crisis.
Environment	Birds tweeting, sun light coming through the window, flower fields, few cars, sunlight on solar panels.

*^1^Plus-buildings are buildings that produces more energy than it uses.*

*^2^Refrigerators with free food soon to expire.*

**FIGURE 3:**
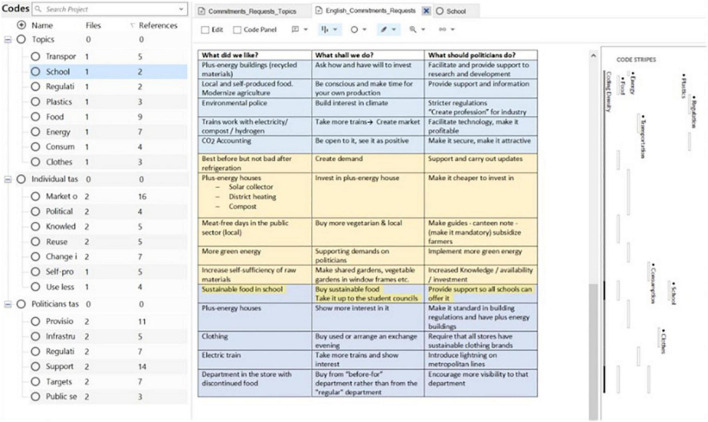
Categorization of tasks from the lists of commitments using software NVivo 20.

When it comes to actions, participants pointed toward changes in behavior and lifestyle, such as traveling by bike or growing food at home and communal sharing. Furthermore, they call on politicians to provide the conditions for those changes to happen based on information and regulations (Table 5). In dissonance to the future stories, school does not occupy a central role in the proposed actions but are mentioned (Table 6). Tasks were transcribed and categorized according to themes (see Figure 3).

**TABLE 5:** Identified topics and actors’ tasks.

Topics	- Clothing – Consumption – Energy – Food –Plastics – Transportation – Regulation – School
Individual tasks	- Changes in behavior - Knowledge sharing - Market offers and demands - Political action - Reuse - Self-production - Use less
Political tasks	- Infrastructure - Provision of information - Public sector actions - Regulations for market - Support mechanisms - Development of targets

**TABLE 6:** Tasks categorized under the topic “School.”

Liked	Individual tasks	Politicians’ tasks
Environment and climate in the school	- Take to student council. - Discuss with teachers. - Discuss among students.	- Include in the curriculum. - More resources to work with and learn about the environment. - Climate and environment as an elective class.
Sustainable food offer	- Buy sustainable food. - Take to student council	- Support sustainable food in school canteens

In the report published after the workshop, there were eight main themes: (1) Transport, (2) Food, (3) Plastics, (4) Clothes and reuse, (5) Waste, (6) Buildings and energy, (7) Care for nature, forests, and woodwork, and (8) Knowledge, awareness, and attitude change (Lorgen and Ursin, 2019). These themes represent concrete actions. The final strategy is more general, focusing on six areas for transformation to mitigate climate change: (1) Buildings, (2) Carbon sequestration, (3) Food, (4) Materials and Plastics, (5) Transport, and (6) Meeting places (Trøndelag County Council, 2020). The first five areas encompass concrete examples proposed by the children and youth. However, the sixth one represents an interest by the county council to open their engagement channels.

The written feedback shows that the participants perceived the Climate Workshop as an initiative that “takes our opinions seriously” (participant feedback), generating ideas of how to prevent the destruction of the earth through everyday changes. Some of the participants highlighted a sense of making an impact, as stated by one participant, “I feel like I have made a difference.” Getting to know young people who share the same passion and interest in climate change and sustainability was also perceived as valuable, and several formed new friendships. One participant said: “We have become a big community.” The feedback also reveals a sense of optimism, stating for instance: “The earth must be saved, and this weekend made me believe that we might succeed. So many are engaged!”

Many participants highlighted that the workshop was educational and that they learned a lot from each other, stating for instance “It has been really fun and educational.” As a youth facilitator explained: “I am really impressed with the level of knowledge and engagement in the group, and I think the activities were good in showing this [their knowledge] and generating ideas for new solutions.” Yet, some participants had wanted more information about the climate crisis in advance, arguing that “[t]hrough inviting experts people get to know the facts and get a better understanding.” Several participants highlighted the value of hearing group members’ perspectives on climate change and mitigation. Some underlined that everyone was invited to speak and that everyone was engaged. As one participant explained: “[The workshop activities] required thinking and not only relaxing, [it] actually got everyone involved and included all our ideas.” Another participant reported: “I learned a lot – since many had different priorities concerning climate. We got to listen to different perspectives.”

Overall, the participatory design of the workshop seems to have been experienced as fun, creative, and meaningful to participants and youth facilitators. The task-based activities were met with enthusiasm as expressed through feedback such as “I liked that we got to be creative through writing, drawing and discussing both today’s problems, possible solutions, and what the future might look like.” One participant commented that “You get to illustrate your thoughts so that it’s easier to others to see what we think.” Some, however, noted that “It was a little hard to be drawing all the time – I’m not that creative.”

## Discussion

Drawing on the workshop material, participants’ and youth facilitators’ feedback, and our own experiences and reflections, this section is divided into five themes: (1) Enabling a sense of citizenship, (2) Generating meaningful conversations and new perspectives, (3) Being creative and producing visual material, (4) Creating a social space of optimism, and (5) Sparking intergenerational power redistribution.

### Enabling a Sense of Citizenship

Participants expressed appreciation for being included and taken seriously, recognizing that the workshop created an arena of political inclusion for them. They also underscored the importance of increased information about climate-related issues in education in the workshop, which suggests a view of young generations as important stakeholders in climate politics and action. In addition, the workshop materials and subsequent report (Lorgen and Ursin, 2019) sent a message to the politicians, bureaucrats, and citizens of the county that the young generation matters. The workshop was an acknowledgment of children’s and young people’s agency, similar to the approach described by Collin and Swist (2016) for using youth’s expertise for campaigns that are directed to youth. Children and youth are typically marginalized in the political sphere (Lorgen and Ursin, 2021), including in climate politics (Percy-Smith and Burns, 2013; Davies et al., 2016). Climate issues are extremely complex and often left for specialists to discuss and address. Some of the young workshop participants expressed similar views, pondering “Why are they asking *us*? We’re not scientists.” However, an increasing number of politicians, policymakers, and researchers are supporting the inclusion of children and youth in politics as part of advancing democracy (see Wall, 2014; Lorgen and Ursin, 2021). As Wall (2014) contends, the views of children and youth in politics will inform and improve decision-making: “Since nobody can rightly claim a monopoly on what is best for groups in society, it is wiser to allow the greatest possible diversity of voices to influence public debate” (p. 114).

In terms of citizenship, inclusion, and democracy, it is important to critically reflect on processes of recruitment and participation. Children and youth are not a homogenous group with one set of agreed upon opinions. The open invitation to an event, free of charge, was meant to ensure the participation of young people independent of gender, ethnicity, and cultural, geographical, and socio-economic background. The county council made a massive effort in facilitating for the participation of all interested, regardless of their geographical location (for example by bringing some participants from remote areas in by taxi). Although geographical diversity was achieved—young people from the whole region participated—the group of participants seemed somewhat homogenous in other ways (socio-economic class, ethnicity, political engagement). We might have achieved a more diverse group by more actively recruiting in schools in more disadvantaged and ethnically diverse urban areas. However, the Climate Workshop was not intended to be a general hearing or referendum. More participants would require a larger budget and more time in addition to a different methodology. The intention of the workshop was rather to provide an opportunity for young citizens to offer their opinions on climate issues. The choice to run the workshop as a two-day event at a conference hotel during a weekend is likely to have appealed to those who had an existing engagement in climate action. Young people are often expected to bring an air mattress, sleep in gyms, and eat cheap food in similar climate initiatives. As many participants expressed in the written feedback, holding the workshop at a hotel represented importance. We therefore underscore the importance of organizing the workshop free of charge and covering costs such as transportation, meals, and hotel.

The involvement and empowerment of children and youth citizens through participatory events also posits a dilemma about their influence on the to-be strategy for climate mitigation and adaptation. Debates around participatory efforts with disempowered citizens are present in planning and public organization literature. For example, Arnstein (1969) proposed a typology of levels of participation to answer the debate on redistribution of power—citizens with no power being under control vs. having control. Furthermore, the real power of children and youth could be undermined by being represented as a community—that appears empowered—while officials hold decision-making authority (a political body in this instance) (Levine, 2017). The question is whether the workshop increased or reduced the process that some children and youth activists had already started by actively engaging in climate protests—to leverage governmental action. Their concerns may not be the same as those purported by the institutions on the governing side (Trøndelag County Council), which could be in part the result of a generational gap or a dissonance of expectations (Angheloiu et al., 2020). However, the workshop was an opportunity to involve children and youth at the grassroots level and the county council as an institution with participatory approaches as intermediation (Teli et al., 2020). In terms of enhancing intergenerational justice, such initiative can be interpreted as an effort to re-distribute intergenerational power and to cater to interests and aspirations of both the lived present and the unknown future. This is of particular importance when we bear in mind the asymmetric independence of interests (Gardiner, 2012/2003) where young people depend on adults’ climate actions; not vice-versa.

In climate politics as in politics in general, adults are perceived as having the necessary maturity and expertise, and they have the duty to protect the rights of children and the unborn (Davies et al., 2016). Cohen (2005), however, questions whether parents represent their children’s interests at the ballot box (that is, whether they know what their children wish and whether this corresponds with their own wishes). Regarding climate action, there is undoubtedly an intergenerational conflict of interests, touching upon vital inter-temporal distributive questions where people must commit to radical change to fulfill their minimal duties of justice vis-à-vis future generations (Meyer, 2012). As decades of environmental politics on local, national, and global levels have demonstrated, there is a general lack of will to pursue policymaking that ensures intergenerational environmental justice. Policies and lawmakers are generally more concerned with present addressees and short-term (often electoral) effects than with the long term (Campos, 2018). According to Birnbacher (2012/2009), although most adults accept future-oriented ethical principles, they compete with other and present-oriented motivations and are less likely to be given priority in concrete practice. To empower children and youth in climate politics can be seen as a way of reducing intergenerational conflict of interests and solving inter-temporal distributive questions, as youth participants in this case envision a future shared with next generations.

### Generating Meaningful Conversations and New Perspectives

The workshop activities were designed to share knowledge, stimulate individual reflection, exchange viewpoints, and shape collective messages. Participants were encouraged not only to share their worries but also their ideas about solutions. The activities intended to encourage reflections and comparisons through exchange of viewpoints and group discussions. This process allowed for different viewpoints to emerge, as participants were faced with each other’s perspectives and had to come to a consensus through collective ranking and visual messages, an aspect appreciated in the participants’ feedback. As one youth facilitator reported, one group had to reach consensus when one participant shared a photo of an avocado to symbolize unnecessary emissions while another shared a photo of vegetables, including an avocado, as an argument for fewer emissions through veganism. The group discussed the complexities of eating climate-friendly food.

The exchange of ideas and opinions invited consideration of familiar problems in a new light, offering new outlooks and insights. This was particularly evident in the encounter between urban and rural participants, as many of the urban activists urged for vegetarianism as an important step to more sustainable living whilst young rural people emphasized the benefits of a local food system, shortening the distance between food producers and consumers and favoring locally produced fruits, vegetables, and meats. Although reaching a consensus could be difficult, the youth facilitators noted that the groups would discuss back and forth before writing anything down, interpreting this as an effort to include everyone in the message conveyed by the group. Age and maturity were raised as a potential barrier of inclusion, where a youth facilitator reported that it was challenging at times to engage the youngest participants in group discussions. Contributing by drawing and writing was helpful in this regard.

The participants’ feedback reveals that most of them learned from each other, an aspect often cherished, while some also missed the opportunity to learn more about climate issues from professionals (see Ursin et al., in review for more). Manzini (2016, pp. 57–58) calls it “participationism” when facilitators do not offer expert perspectives. In some cases, this could hinder knowledge exchange. The process of developing the strategy reveals the tensions between public and expert knowledge: One of the difficulties that participatory methods in design seeks to resolve is utilizing and integrating a diverse knowledge base in seeking solutions. While expert knowledge is legitimate, for example scientific knowledge on climate change, this knowledge does not consider the implications for different groups of people and their lived experiences (DiSalvo et al., 2012). Nevertheless, the workshop is a co-optation of children and youth’s knowledge by institutional means—at the risk of erasing the contestation and adversarial nature of the original protests. Yet, as Teli et al. (2020) recommend, the participatory design process should look at follow-up and methods as actions for making the future. Furthermore, the workshop’s success should not be measured by the knowledge produced but by the new conversations between previously unrelated actors in this instance, opening up for an intergenerational dialogue.

The intention in gathering public knowledge is to identify gaps between the pathways proposed by experts and what participants desire to be put forward. An ethical principle that applies here, as noted by Robertson and Wagner (2012, p. 65), is respect for (young) people’s expertise. In the case of children and youth, this means elucidating what is understood or imagined about climate change, how it is encountered in everyday life, and the actions that are expected. As Qvortrup (2009) underscores, children as a generational category might have different priorities than adults. This is particularly relevant in climate politics, where the youngest generation are most vulnerable to climate change and climate induced effects and will bear the brunt of the impacts of long-term climatic changes (UNICEF, 2008; Davies et al., 2016). For instance, while experts could be setting their hopes on individually owned electric vehicles, the young participants leaned toward public transportation by combining the use of publicly owned bikes and other modes of transit such as trains and buses. Although the county council originally showed skepticism toward the open-ended participatory design, resting solely on the input of the participants rather than lectures being part of the event, they were pleasantly surprised by the richness of material that the workshop generated. An open-ended approach also guaranteed legitimacy of the final report, as participants had not been influenced by other stakeholders in the process (see also Punch, 2002).

### Being Creative and Producing Visual Material

**PHOTO 4:**
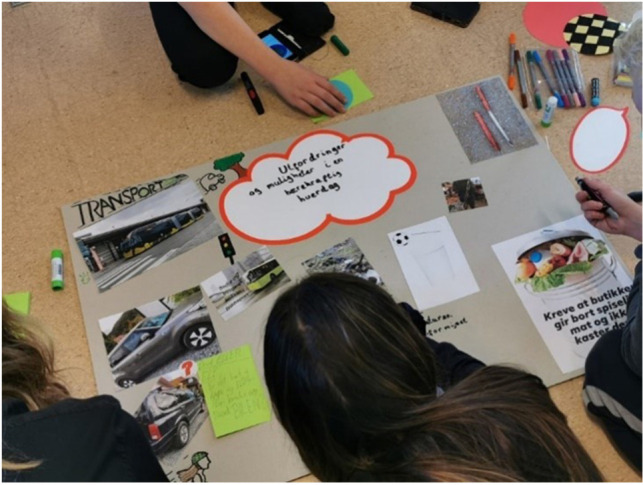
Participants utilizing the workshop space.

The workshop activities encouraged various forms of expression, including discussion, writing, drawing, and using photos and news clippings to convey messages. A youth facilitator expressed being impressed by the workshop design, noting that it “felt like something different than just another workshop.” Task based activities can also allow more freedom of movement than for example interviews, potentially contributing to an atmosphere that is comfortable, yet dynamic and active. As we moved from the first initial discussions to task-based activities, participants began to engage more with each other and ‘took over the room’ by utilizing the space in different ways, some of them spreading out on the floor, making posters (see Photo 4). However, tasks were time-consuming, and the tight time schedule presented a challenge for both participants and the research team throughout the weekend.

The workshop was perceived as fun and met with enthusiasm. Some also found that the visual tools eased the process of communication, overcoming the logocentric tendencies of talk, as one participant noted, “You get to illustrate your thoughts so that it’s easier to others to see what we think.” According to Elden (2012), to communicate ideas visually enables the abstract to become concrete. In agreement with Buckingham (2009), we do not see some methods as offering “privileged access to what people ‘really’ think or feel” (p. 635). However, we see benefits in drawing on a ‘tool kit’ of various methods as a way of making research understandable and to aid in acknowledging different preferences and abilities (Ennew and Plateau, 2004). This helps reposition children and youth in policymaking in climate politics (cf. Gardiner, 2012/2003; Davies et al., 2016) and ensure their participatory rights as they may provide their opinions (United Nations, 1989, Article 12) by using a medium of their own choice (Article 13). Some also found it challenging to be visual and creative; some participants felt they lacked artistic competence and may feel constrained and uncomfortable with methods like drawing (Punch, 2002; Buckingham, 2009). We aimed at allowing flexibility in how tasks were solved by inviting participants to choose means of expression. However, as preferences vary from person to person, it is challenging to create a workshop design that accommodates all. The event was open to children and youth of different ages, levels of knowledge, and political engagement, making it a challenge to balance; acknowledging different preferences and competencies while not being patronizing (Punch, 2002) or homogenizing children and youth (Thomson, 2007).

Visual and creative tasks can be seen as less political. Some participants expressed a wish for more “actual politics” and debate in a more traditional manner. In the process of writing this article, we reflected on how the task of creating a positive story about the future may be experienced as slightly belittling and an obstacle to political involvement. Being invited to create a story about a *fictional* character in the future can be experienced as being asked to write ‘make-believe stories’ rather than dealing with political questions (see Ursin et al., in review). Yet we used this method as a means for participation and an effective way of envisioning futures that engenders ethical questions (Baumer et al., 2018). The creation of stories engages the imagination of participants in a (politically) enabling way (see Borup et al., 2006). The future workshops method is commonly used with adult participants. However, considering the potential sensitivity of the activity, when working with children and young people, communicating the reasons for using this method and its political dimensions in a clear manner is important.

**PHOTO 5:**
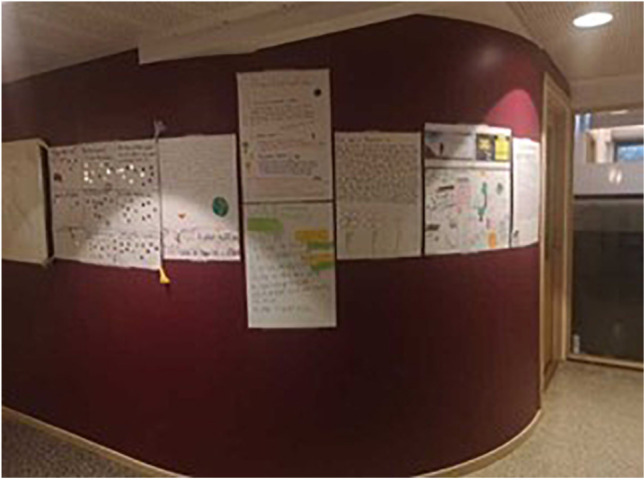
Materials displayed in ‘corridors of power.’

**PHOTO 6:**
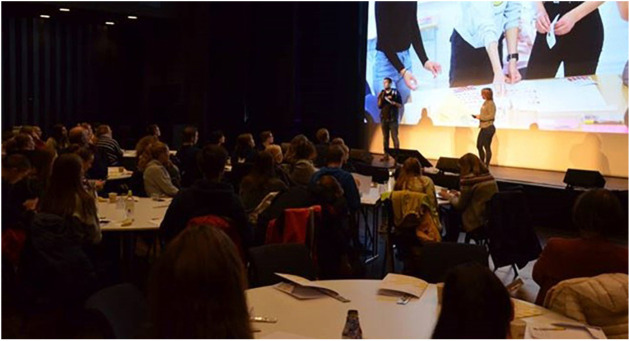
Presentation of input from the Climate Workshop at EnergyChange conference 2019 in Trondheim.

The task-based and visual methods produced visual outcomes that are useful as research material and boundary objects in discussions with other groups or communities around the same topic (Ehn, 2008). Immediately after the workshop, the visual material (posters and collages) was displayed in the hallway outside the political and administrative wing in the county municipality hall as a teaser before the report was published (see Photo 5). Through this, the voices of children and youth were made visible in the ‘corridors of power,’ carrying substantial symbolic meaning. The inputs – in form of photos taken at the event, the physical posters and collages made by the participants and the final report (Lorgen and Ursin, 2019) – became the foundation for the succeeding workshops and conferences held with different groups of (adult) stakeholders (see Photo 6). The visual material turned out to provide a good angle from which to look at the specific inputs and worked as icebreakers and conversation catalysts resulting in meaningful conversation.

### Creating a Social Space of Optimism

The social dimension of the Climate Workshop was appreciated by the young participants who enjoyed the opportunity to socialize with like-minded and establish new friendships. Some participants arrived alone, others with friends. They socialized through workshop activities as well as during breaks, meals, and other social gatherings throughout the weekend. Being a young activist can be a lonely experience (Hondsmerk, 2021), particularly in small towns. In addition, psychologists are becoming increasingly concerned about the strain the climate crisis is putting on young people’s mental well-being and report environment-related stress and anxiety (i.e., Clayton et al., 2017; Clayton, 2020; Skauge and Haugestad, 2020). Promoting a sense of connectedness with others through climate action is vital in reducing climate anxiety (Clayton et al., 2017; Skauge and Haugestad, 2020; Hondsmerk, 2021).

The workshop also fostered a sense of optimism, a feeling of “having made a difference” (participant feedback). One youth facilitator reflected on a shift from a pessimistic to a positive tone when participants were made aware that the future visions task was to be an optimistic portrayal. The workshop results were permeated with anticipation: Participants envisioned radical change and increased life quality of citizens (Lorgen and Ursin, 2019). The initiative thus had outcomes beyond the democratic and political intention, nurturing a sense of well-being among the young participants. Participants expressed a belief that a society with substantially lower environmental impact is a better one, in terms of life quality, solidarity, and health. They imagined a green society marked by biodiversity (birds tweeting, flowers in the city, few cars), where we eat locally produced food (homegrown in windowsills or roof greenhouses), our transit options are smart (electric bicycles, electric buses and trains and flying buses), our buildings are climate friendly (solar panels and plus-buildings), and our habits and behavior are focused on sharing, repairing, and being together. Indeed, when activism cultivates a sense of meaning and purpose, active engagement in efforts to mitigate climate change is reported to reduce feelings of fatalism, helplessness, hopelessness, and lack of understanding (Clayton et al., 2017; Clayton, 2020; Hondsmerk, 2021).

From an ethical perspective, we were wary of the risks of causing emotional distress or environment-related anxiety among our participants (Clayton et al., 2017; Clayton, 2020). In addition, we wondered whether it is fair to ask children and youth about solutions on complicated issues that they are not responsible for. As elsewhere, young Norwegians engaged in climate activism have adopted a common identity as ‘the future’ and report higher environment-related stress than older generations (Skauge and Haugestad, 2020). Although one might ask whether young people embrace this label of futurism as a response to policy instruments that hinge on the planetary legacy for ‘future generations,’ it is also worth considering whether such initiatives further chisel out their status as agents of change (see Walker, 2017), making them responsible for the mistakes of previous generations. Making matters worse, due to their intergenerational positioning, children and youth have little real political agency (Walker, 2017; Ursin and Lorgen, 2019) and their participation in environmental politics remains “naïve, simplistic and tokenistic” (Percy-Smith and Burns, 2013, p. 324).

These concerns suggest a need for careful consideration of methodological choices to help ameliorate distress. In the Climate Workshop, the activities focused on a positive future, and areas and actions of improvement. As such, they were imbued with anticipation, hope, and optimism. As recommended by the American Psychological Association, to promote resilience in the face of the climate crises, the workshop also brought young people together for mutual support and provided opportunities for meaningful action (APA as cited in Clayton et al., 2017). Furthermore, to be ethical, research must be of sufficient importance, and the benefits must outweigh the risks, ensuring participants’ rights to be protected from exploitation (United Nations, 1989, Article 36) (Ennew et al., 2009). As the results from the workshop informed the region’s strategy for transformations to mitigate climate change, it can be argued that the participation of children and youth as representatives of the future (Weston and Bach, 2009) leverages their positions at the margins of political arena, this outweighing potential risks of causing distress and anxiety. Furthermore, their cross-temporal position renders climate mitigation as of particular importance to them, as they may live longer and experience the birth of their children and grandchildren.

This might suggest that children and young people are less concerned with short-term investments and politics and more prone to embrace environmental issues and the well-being of future generations. As in the words of climate activist Hondsmerk (2021), young activists are willing to commit civil disobedience and even get arrested for future generations. This fits well with Rawls’ 2012/1999 ‘chain of concern model,’ where action promotes indirect future-oriented reciprocity (Rawls as cited in Weston and Bach, 2009). The young participants called for the need for environmental police and legal sanctioning of climate offenses and showed great concern for biodiversity, calling for the protection of all species’ habitats, for instance by cleaning plastic from the ocean. Their attitude is aligned with a respect-based intergenerational justice, based on the idea of a transgenerational and transtemporal global social contract founded on the notion of human and non-human solidarity (see also Campos, 2018).

### Sparking Intergenerational Power Redistribution

The young Swedish climate activist Greta Thunberg provided young activists new legitimacy in raising their voice and criticizing the neglect of environmental concerns in decision-making, both related to industries and politics, demanding a more rapid and transformative change. Inspired by Greta Thunberg and her followers, young people in Trøndelag mobilized through school strikes organized by the global ‘Fridays for future’ movement. The politicians in the County Executive Board of Trøndelag expressed a wish to understand the underlying motivations of the youth climate strike. The Climate Workshop was an opportunity to empower the perspective of these already active participants and inform public and politically elected authorities, influencing the making of a new strategy to mitigate climate change. However, participation is not inherent to research methods (Thomson, 2007), and organizing a participatory workshop does not guarantee real participation in policymaking. To ensure participatory rights of children and youth, their views must be given due weight (United Nations, 1989). In addition, any participatory process should ensure that the solutions put forward are for the benefit of all affected groups. This requires a political commitment toward enacting and inspiring social change and challenging unequal power relations (Grant, 2017). Initiatives where children and youth are consulted but not taken seriously are tokenistic, a form of non-participation in decision-making (Lundy, 2018).

The process from the Climate Workshop until the final strategy shows how policymakers addressed issues raised by the young participants and demonstrates that their views were taken seriously. Crucial was the *timing* of the event. The Climate Workshop was held early in the political process of developing a climate mitigation strategy (see Figure 1), which enabled children’s and youth’s perspectives to form a *foundation* rather than a *supplement* to the resulting policy. The young people clearly stated that the solution to the climate crisis lies in cross-sectoral solutions, where various actors in society work together to achieve the goal of a net-zero society. Their input followed the rest of the process of making the strategy in various ways. The initiative was partly *youth-led* (see Landsdown, 2010) as it originated through young people’s public protests and social mobilization, inspiring the county council to invite young people to share their opinions. Members of the youth county committee were consulted throughout the process, and their views influenced the final strategy. In the process, various groups had the opportunity to voice opinions, including youth operating within the political system, young activists from outside the political establishment, and children and youth who were not organized or formally politically active, but engaged in the issue.

Although workshop participants were homogenous in some regards, a heterogeneity of young voices was thereby included, which in our view strengthened the knowledge foundation produced. Trøndelag County Council has institutionalized youth involvement through the youth county committee, which can influence policymaking and make recommendations to politicians. Both the youth county committee and local environmental organizations participated in the planning of the workshop and reviewed the ways in which the results were present in the final strategy, strengthening the quality of the workshop and the process before and after it. However, young people were not involved in all aspects of planning and carrying out the workshop (primarily done by researchers and administration in county council) and implementing the results into the final strategy (decided by county council members). As such, the Climate Workshop was situated in the nexus between consultative and collaborative participation (Landsdown, 2010), sharing views and ideas in an adult-led and managed event and influencing the process, but simultaneously being excluded from decision-making processes. This was, however, also the case for other interest groups such as researchers and adult stakeholders.

The climate transition strategy of Trøndelag (Trøndelag County Council, 2020) is based on input from the report on Climate Workshop in addition to knowledge from international climate research and national expert papers on how to tackle climate change in Norway. It may be hard to discern the actual impact of the Climate Workshop as the workshop inputs are overlapping with priorities of experts on climate mitigation. The emphasis on materials and plastic, however, undoubtedly stems from youth engagement. A divergence between the youth’s wishes and demands and the final strategy concerns time. The solutions and timeframe proposed do not meet the youth participants’ expectations in terms of time and radicality of societal changes. Despite a joint goal, the timing of crossing the finish line is significantly later in the final strategy than it would be had it been up to the youth. The outcome of the Climate Workshop thus suggests that there is a divergence in what the youngest generation perceives as ‘just savings’ for generations to come (cf. Rawls, 2012/1971) and what the adult population is willing to do.

The photos and illustrations from the Climate Workshop in the strategy document situates young people visually at the heart of the strategy. However, this also raises critical and ethical questions, such as whether it leads to an exaggerated impression of their inclusion in the political process. One can ask if the strong visual position of children and youth is a rhetorical utilization of their symbolic power. Children and young people embody our perception of ‘the next generation,’ a symbolic evocation of hope, futurity, and social change, that commonly calls for concerted public and political action on climate change (Walker, 2017). However, as Walker (2017) points out, the use of children and young people as symbols of change is inherently problematic when they are seen as citizens-in-the-making (Lorgen and Ursin, 2021), marginalized in decision-making processes.

In addition to having an impact on the climate strategy, the Climate Workshop and the subsequent report affected the work of the youth county committee. It strengthened the competence, capacity, and awareness related to climate change transformation in the committee, making climate transition one of four action areas in their yearly work plan. The committee has also worked on several projects related to the Climate Workshop, such as the production of an informative video about how youth can make their municipality help fight climate change. They also informed the President of the Parliament about the workshop and the importance of including youth in decision-making related to climate change and inspiring youth across the country to demand climate action (UFT/UFU, 2020).

In retrospect, we realize that one area of improvement is the structure of feedback sent to the youth participants. The participants received a newsletter specially made for them. However, since politicians ordered the workshop and asked the young people to contribute, feedback from these politicians on how the input was received and implemented would have been preferable. This would have provided transparency in the decision-making process and encouraged accountability, conveying the message that youth’s suggestions were or would be implemented (see Lundy, 2018). In addition, the material is co-produced knowledge, thus the youth participants could have received a summary of the raw material of the workshop to increase their sense of ownership, allowing them to use it (e.g., showing it to family and friends, presenting it in school, using it in organizational work, etc.). This might also have led to amplified effects of power redistribution.

Lastly, a common criticism of participatory design methods is that participation is reduced to administrative—one time—events that undermine the possibility for long time committed interactions between multiple interested parties (Botero and Hyysalo, 2013; Manzini, 2016). The current process was limited in the sense that it did not result in multiple iterations, however, the current climate strategy is not fixated on specific solutions. This is an opportunity for young people to articulate their participation even more by putting visions into concrete solutions. While Trøndelag County Council intents to mediate participation, it is unclear how this will occur.

## Conclusion

Children and youth hold a vital position in climate politics and are perhaps the most important stakeholders. They hold a key position in sustainable politics, as Heft and Chawla (2006) point out, “if practices consistent with sustainable development are to be carried forward through time, then children must be the bridge conveying their value and ways” (p. 199). Based on our experiences with the Climate Workshop, we propose that participatory workshops, focusing on intertemporal aspects and the (desired) future of the participants (Jungk and Müllert, 1997), may ensure their participatory rights and enhance their sense of citizenship as well as strengthen intergenerational justice by a redistribution of power in the present. In addition, such initiatives provides intergenerational perspectives and reduces the intergenerational gap. There is, however, a need for longer term participation with children and youth, both to foster a sense of ownership and to ensure continuity for their visions (Botero and Hyysalo, 2013; Teli et al., 2020). Therefore, we suggest that such workshops become permanent mechanisms of citizen participation in decision-making in community development to recognize and protect the human rights of present and future generations.

## Data Availability Statement

The original contributions presented in the study are included in the article/supplementary material, further inquiries can be directed to the corresponding author/s.

## Ethics Statement

Ethical review and approval was not required for the study on human participants in accordance with the local legislation and institutional requirements. Written informed consent to participate in this study was provided by the participants’ legal guardian/next of kin. Written informed consent was obtained from the individuals and minors’ legal guardian for the publication of any potentially identifiable images or data included in this article.

## Author Contributions

MU, LL, and IA developed the methodology and design of the Climate Workshop and have contributed to sections about “Materials and Methods,” “Results,” and “Discussion.” A-LS, RN, MB, and KB have contributed with their perspectives on the challenges and strengths of the workshop and its role in the shaping of Trøndelag County Council’s strategy for climate mitigation. MU has elaborated the theoretical framework on intergenerational justice and climate legacy and has coordinated the final manuscript as a whole. All authors have made a substantial contribution to the manuscript, read and agreed to the publication of the contents of the manuscript.

## Conflict of Interest

The authors declare that the research was conducted in the absence of any commercial or financial relationships that could be construed as a potential conflict of interest.

## Publisher’s Note

All claims expressed in this article are solely those of the authors and do not necessarily represent those of their affiliated organizations, or those of the publisher, the editors and the reviewers. Any product that may be evaluated in this article, or claim that may be made by its manufacturer, is not guaranteed or endorsed by the publisher.

## Footnotes

1 Ursin, L., Alvarado, S., and Nordgåar (in review). “Children and youth participation in climate policy: a dialogue beyond the workshop,” in *A New Handbook of Children and Young People’s Participation: Conversations for Transformational Change*, eds B. P. Smith, N. P. Thomas, C. O’Kane, and A. T.-D. Imoh (Abingdon-on-Thames: Routledge).
